# Does the use of the 2009 FIGO classification of endometrial cancer impact on indications of the sentinel node biopsy?

**DOI:** 10.1186/1471-2407-10-465

**Published:** 2010-08-30

**Authors:** Marcos Ballester, Martin Koskas, Charles Coutant, Elisabeth Chéreau, Jeremy Seror, Roman Rouzier, Emile Daraï

**Affiliations:** 1 Service de Gynécologie-Obstétrique, hôpital Tenon, AP-HP, CancerEst, Université Pierre et Marie Curie, Paris VI, France

## Abstract

**Background:**

Lymphadenectomy is debated in early stages endometrial cancer. Moreover, a new FIGO classification of endometrial cancer, merging stages IA and IB has been recently published. Therefore, the aims of the present study was to evaluate the relevance of the sentinel node (SN) procedure in women with endometrial cancer and to discuss whether the use of the 2009 FIGO classification could modify the indications for SN procedure.

**Methods:**

Eighty-five patients with endometrial cancer underwent the SN procedure followed by pelvic lymphadenectomy. SNs were detected with a dual or single labelling method in 74 and 11 cases, respectively. All SNs were analysed by both H&E staining and immunohistochemistry. Presumed stage before surgery was assessed for all patients based on MR imaging features using the 1988 FIGO classification and the 2009 FIGO classification.

**Results:**

An SN was detected in 88.2% of cases (75/85 women). Among the fourteen patients with lymph node metastases one-half were detected by serial sectioning and immunohistochemical analysis. There were no false negative case. Using the 1988 FIGO classification and the 2009 FIGO classification, the correlation between preoperative MRI staging and final histology was moderate with Kappa = 0.24 and Kappa = 0.45, respectively. None of the patients with grade 1 endometrioid carcinoma on biopsy and IA 2009 FIGO stage on MR imaging exhibited positive SN. In patients with grade 2-3 endometrioid carcinoma and stage IA on MR imaging, the rate of positive SN reached 16.6% with an incidence of micrometastases of 50%.

**Conclusions:**

The present study suggests that sentinel node biopsy is an adequate technique to evaluate lymph node status. The use of the 2009 FIGO classification increases the accuracy of MR imaging to stage patients with early stages of endometrial cancer and contributes to clarify the indication of SN biopsy according to tumour grade and histological type.

## Background

Introduction of a new cancer classification is required when sufficient evidence-based data proved their impact on the staging itself. Recently, the scientific community with the support of the Federation International of Gynecology and Obstetrics (FIGO) as well as other international scientific societies and agencies has considered that revision of the classification of endometrial cancer was necessary. This was mainly based on data showing that the prognosis of stage IA grade 1-2, and IB grade 1-2, had similar 5-year survival. Based on the favourable prognosis for the former stage IA and IB patients, the FIGO Committee elected to merge these stages so that now stage IA involves the endometrium and/or less than one-half myometrial invasion and IB is equal to or greater than the outer one-half of the myometrium [1-3].

The impact of a new classification implies that studies using the former classification become obsolete rending necessary temporally to express data using the two classifications to adapt therapeutic strategy. This is particularly important concerning the requirement of lymphadenectomy that is associated with morbidity in women with endometrial cancer that are often obese with hypertension and diabetes. Since 1988, FIGO classification ruled on surgical staging of endometrial cancer, based on peritoneal cytology, hysterectomy, bilateral salpingo-oophorectomy and pelvic/paraaortic lymphadenectomy [4]. Previous studies attempted to identify patients that could benefit of lymphadenectomy according to presumed FIGO stage assessed by imaging techniques, histological type and histological grade obtained on biopsy [5]. Recent randomized trials have suggested that lymphadenectomy has little relevance on survival and can be omitted in early stages of endometrial cancer [6]. These results were based on routine histology with hematoxylin and eosin staining (H&E) without the use of neither sentinel node procedure nor serial sectioning and immunohistochemical staining. Hence, it is likely that some patients are under staged linked to misdiagnosis of micrometastases [7-9]. Although there is no consensus on sentinel node biopsy neither on the prognostic relevance of micrometastases in endometrial cancer, Yasbushita et al demonstrated the relation between the presence of micrometastases and the risk of recurrence [10]. Therefore, the aims of the present study were to evaluate the relevance of the sentinel node (SN) procedure in women with endometrial cancer and to discuss whether the use of the 2009 FIGO classification could modify the indications for SN procedure.

## Methods

### Patients

Between July 2002 and March 2009, 85 women with suspected endometrial cancer were referred to the gynecology unit of Tenon Hospital, Paris, France. Among the 85 women, results of the first 46 women have been previously published [7]. All the women underwent endometrial biopsy under hysteroscopy guidance to diagnose cancer and preoperative MR imaging to assess the disease stage. All the women underwent peritoneal cytology, the SN procedure with pelvic lymphadenectomy, bilateral salpingo-oophorectomy and hysterectomy by laparoscopy. Women with presumed involvement of cervical stroma on MR imaging had radical hysterectomy instead of simple hysterectomy.

Medical records were reviewed to determine age, body mass index (BMI), tumour stage, histology, surgical procedure, intra- and postoperative complications, and length of hospital stay. Outcome was obtained from the outpatient records. The study was approved by the institutional review board (CEROG-2010-34) and all patients provided written informed consent.

### Sentinel Node Procedure Technique

The sentinel node procedure technique was performed as previously described [7]. In summary, four intracervical injections of 0.2 mL of unfiltered technetium were administered the day before surgery. Scintigraphic images were obtained 2 h after the injections and then every 30 min to detect the sentinel node (SN).

Before starting surgery, under general anaesthesia, patent blue was injected intracervically at 3 and 9 h clock. The pelvic and lower para-aortic regions were inspected carefully for lymph ducts and dye uptake by lymph nodes. Radioactive pelvic and para-aortic lymph nodes were located by using an endoscopic gamma probe (Eurorad, Strasbourg, France) inserted through the 12-mm suprapubic trocar.

Radioactive lymph nodes were sought before opening the peritoneum. After locating the SN, the peritoneum was opened and each blue and/or radioactive lymph node was removed separately in endoscopic bags. Pelvic lymphadenectomy was systematically performed after the SN procedure. All lymphatic tissue was removed and extracted in an endoscopic bag. Women with clear-cell or serous endometrial cancer had systematic omentectomy with para-aortic lymphadenectomy. Usual boundaries of para-aortic lymphadenectomy were respected with the left renal vein as the upper limit. The absence of residual pelvic or para-aortic radioactivity was verified after lymphadenectomy.

### Histology

SN and non-SN were analyzed by a pathologist. Lymph nodes with macroscopic metastases were sectioned. Normal-appearing SNs were cut perpendicular to the long axis. All SN were submitted to intraoperative imprint cytology. Air-dried cytological smears were prepared by scraping the cut surfaces and staining with a rapid May-Grünwald-Giemsa method. Each half-SN was sectioned at 3-mm intervals. Each 3-mm section was analyzed at four additional levels of 150 *μ*m and four parallel sections; one was used for H&E staining, and H&E-negative sections were examined by immunohistochemistry (IHC) with an anticytokeratin antibody cocktail (cytokeratins AE1-AE3; Dako Corporation, Glostrup, Denmark). Non-SN were submitted totally and blocked individually after 3-mm sectioning and H&E staining.

The size of lymph node metastases was estimated with an eyepiece micrometer. A micrometastasis was defined as a single focus of metastatic disease per lymph node, measuring no more than 2 mm. The presence of single noncohesive tumour cells was recorded as submicrometastasis [7,11,12] SNs were considered positive when they contained macrometastase, micrometastase, or submicrometastase.

### Analysis of SN

SNs were recorded as blue-stained and/or radioactive (if the in vivo count exceeded three times the background). The false-negative rate was defined as the number of procedures with a negative SN and one or more positive non-SN, divided by the number of procedures with any positive pelvic or para-aortic node.

### Adjuvant Therapy after Surgery

The 1988 FIGO classification was used in the decision making process for adjuvant therapy after surgery [4]. Adjuvant therapy was not recommended for women with stage IA disease, regardless of the grade. Women with stage IB, IC, IIA or IIB disease had brachytherapy. Women with stage III disease or higher had external pelvic radiotherapy.

Brachytherapy consisted of 20 Gy given 5-6 weeks after surgery. External pelvic radiation therapy consisted of 40 Gy, divided into 2.25 Gy per fraction, four days a week. All fields were treated daily with 15 megavoltage units. When concurrent chemoradiotherapy was indicated, chemotherapy was given during the first and fourth weeks of radiation therapy and consisted of a continuous 5-fluorouracil infusion (750 mg/m2/day), and a cisplatin bolus (20-25 mg/m2/day) 1 h before radiotherapy, on days 1, 2, 4 and 5. Patients with positive aortic nodes received extended-field radiation up to the level of T12-L1.

### FIGO staging

Using the 1988 FIGO classification, presumed stage before surgery was assessed for all patients based on MR imaging features. Then, after surgery and definitive histology, the final FIGO stage was determined. For each patient, the same analysis was performed using the 2009 FIGO classification.

### Statistical Analysis

Statistical analysis was based on Student's t-test and the Mann-Whitney test for parametric and nonparametric continuous variables, respectively, and the chi-square test or Fisher's exact test, as appropriate, for categorical variables. The Mc Nemar test for paired samples was used to compare the detection methods. Values of p < 0.05 were considered to denote significant differences.

## Results

### Epidemiological and preoperative characteristics of the population (Table 1)

**Table 1:** Epidemiological and histological characteristics of the 85 patients with endometrial cancer.

Characteristics	Patients n = 85
Age	66 (range 43-87)

Hypertension	34 (39%)

Hormone replacement therapy	21 (24%)

Obesity (BMI ≥ 30)	19 (22.3%)

Histology:	
Endometrioid adenocarcinoma	73 (86%)
Adenosquamous adenocarcinoma	1 (1%)
Serous papillary adenocarcinoma	5 (6%)
Clear cell adenocarcinoma	5 (6%)
Carcinosarcoma	1 (1%)

Preoperative histological grade on biopsy	
1	41 (48.2%)
2	22 (25.8%)
3	11 (12.9%)
NP	11 (12.9%)

Median age was 66 years (range: 43-87 years). Eighty-one patients (95%) were menopausal, of whom twenty-one (24.7%) had hormonal replacement therapy. The median body mass index (BMI) was 26.5 kg/m^2 ^(range: 17.8-45.3 kg/m^2^). Nineteen patients (22.3%) were obese (BMI > 30 kg/m^2^). Ten patients had a history of breast cancer but none of them underwent a tamoxifen treatment. None of the patients had a history of hereditary non-polyposis colorectal cancer (HNPCC) syndrome.

All patients had uterine bleeding investigated by hysteroscopy and biopsy. Endometrial biopsy showed endometrioid adenocarcinoma, serous papillary carcinoma, clear-cell adenocarcinoma, carcinosarcoma and adenosquamous carcinoma in 73 cases, 5 cases, 4 cases, and one case each, respectively. The tumor grade on biopsy was 1, 2, 3 and undetermined in 41 cases, 22 cases, 11 cases and 11 cases, respectively.

### SN Procedure

The SN procedure was performed by laparoscopy and laparotomy in 79 and 6 cases, respectively. A laparotomic SN procedure was used initially in four patients with a history of pelvic surgery, and conversion to laparotomy was required in two cases because of severe obesity. Seventy-five patients (88.2%) had pelvic lymphadenectomy and ten patients (11.8%) had both pelvic and paraaortic lymphadenectomy for clear cells adenocarcinoma and serous adenocarcinoma in 5 cases each. The SN procedure involved dual and single labeling in 74 and 11 cases, respectively. None of the women had radioactive labeling alone. An SN was detected in 88.2% of cases (75/85 women). The SN detection rates with dual and single labeling were 91.9% and 63.6% (p = 0.01), respectively. A mean (± standard deviation, SD) of 2.6 ± 1.2 and 1.8 ± 0.5 SN per patient were detected with the dual and single labels, respectively (p = 0.2).

The total number of removed pelvic nodes was 1105 (median: 13, range: 2-26) including SN. The total number of right and left external iliac nodes, excluding SNs, were 470 (median: 5, range: 1-13) and 435 (median: 5, range: 1-13), respectively. The median number of para-aortic nodes was 15 (range: 5-26). Among the 75 patients with at least one identified SN, the mean number of SN per patient was 2.68 (range: 1-7). A total of 201 SN were removed. The SNs were blue and radioactive, radioactive alone and blue alone in 105, 96 and 10 cases, respectively. SNs were detected bilaterally in 49 (57.6%) of the 85 patients. The SNs were located in the region of the external iliac (lateral group), the iliac vessel bifurcation, the common iliac and the aortic bifurcation in 160 (78%), 33 (16%), 7 cases (6%) and 1 case (1%), respectively. No SNs were detected in the parametrium or para-aortic area. The patient with an SN at the aortic bifurcation had two SNs in the external iliac region. None of the patients had an isolated paraaortic SN.

### Histology of sentinel and non sentinel nodes (Table 2)

**Table 2:** Tumour characteristics and MRI staging in patients with positive SNs.

Patient	MRI 1988 FIGO stage	MRI 2009 FIGO stage	Definitive Histology	Tumor grade	H&E staining	IHC
**1**	IC	IB	adenosquamous	1	Negative	Positive

**2**	IC	IB	Endometrioid	3	Negative	Positive

**3**	IC	IB	Endometrioid	2	Positive	

**4**	IC	IB	Serous papillary	1	Positive	

**5**	IC	IB	Endometrioid	3	Negative	Positive

**6**	IC	IB	Endometrioid	2	Positive	

**7**	IIA	IA	Endometrioid	2	Positive	

**8**	IB	IA	Clear cells	2	Positive	

**9**	IC	IB	Endometrioid	2	Positive	

**10**	IC	IB	Endometrioid	1	Negative	Positive

**11**	IC	IB	Serous papillary	1	Positive	

**12**	IC	IB	Clear cells	3	Positive	

**13**	IB	IA	Endometrioid	2	Negative	Positive

**14**	IIIA	IIIA	Clear cells	3	Positive	

MRI: Magnetic Resonance Imaging; IHC: immunohistochemistry

Fourteen (18.6%) of the 75 patients in whom at least one SN was detected had a positive SN, with macrometastasis in three cases and micrometastasis in eleven cases. The median number of SN per patient was 3 (range: 1-7). The median number of non-SN removed per patient was 10.5 (range: 2-26). Intraoperative imprint cytology confirmed two of three macrometastasis. SN micrometastasis and macrometastasis was diagnosed postoperatively by H&E staining in seven women. All SNs that were negative by H&E staining were examined by immunohistochemistry (IHC), and seven additional patients were found to be positive. A total of 895 pelvic non-SNs were removed. Thirteen of the fourteen patients with positive SNs had no positive non-SN, while a patient with positive SNs (a macrometastasis and isolated tumour cells) had two positive non-SNs. None of the patients with no positive SNs had a positive non-SNm i.e. there were no false-negative case. None of the ten patients in whom no SN was detected had a positive non-SN.

### Correlation between MR imaging staging and definitive histology (Table 3)

**Table 3:** Relation between presumed MR imaging stage of endometrial cancer and final stage after definitive histology according to 1988 FIGO classification.

FIGO stage after definitive histology							
**Presumed** **Stage on MRI**	**IA**	**IB**	**IC**	**IIA**	**IIB**	**IIIA**	**Total**

**IA**	28.5%	43%	14.2%	0	14.2%	0	14
**IB**	31.5%	40%	23%	5.5%	0	0	35
**IC**	0	18.5%	74%	3.7%	0	3.7	27
**IIA**	0	67%	33%	0	0	0	3
**IIB**	0	50%	0	0	0	50%	2
**IIIA**	0	0	100%	0	0	0	1
**Total**	18.3%	34%	39%	3.5%	2.5%	2.5%	82

Kappa test = 0.24 (moderate)

Using 1988 FIGO classification, MR imaging detected an endometrial cancer of stage IA, IB, IC, IIA, IIB or IIIA disease in 14 cases (17.3%), 35 cases (43.2%), 27 cases (33.3%), 3 cases (3.7%), 2 cases (2.5%) and 1 case (1.1%), respectively. Three patients were not staged on MR imaging.

When considering myometrial invasion using 1988 FIGO classification, among the fourteen women with presumed stage IA disease on MR imaging, final histology showed FIGO stage IA, IB, IC and IIB disease in 28.5%, 43%, 14.2% and 14.2%, respectively. Among the 35 women with presumed stage IB disease on MR imaging, final histology showed FIGO stage IA, IB, IC and IIA disease in 31.5%, 40%, 23% and 5.5%, respectively. Among the 27 women with presumed stage IC disease on MR imaging, final histology showed FIGO stage IB, IC, IIA and IIIA disease in 18.5%, 74%, 3.7% and 3.7%, respectively. Among the three women with presumed stage IIA disease on MR imaging, final histology showed FIGO stage IB and IC disease in 67% and 33%, respectively. Among the two women with presumed stage IIB disease on MR imaging, final histology showed FIGO stage IB, IIB disease in 50% each. The only woman with presumed stage IIIA on MR imaging had a stage IC on final histology. Therefore, 38 of the 82 women (47.5%) were correctly staged by MRI. The correlation between preoperative MRI staging using 1988 FIGO classification and final histology was moderate (Kappa = 0.24)

### Impact of new FIGO classification (table 4)

**Table 4:** Relation between MR imaging stage and FIGO stage at final histology according to the new classification.

FIGO stage after definitive histology					
**Presumed** **Stage on MRI**	**IA**	**IB**	**II**	**IIIA**	**Total**

**IA**	73%	19.2%	7.8%	0	52
**IB**	18.5%	74%	3.7%	3.7%	27
**II**	50%	0	50%	0	2
**IIIA**	0	100%	0	0	1
**Total**	53.5%	37.8%	7.3%	1.4%	82

Kappa test = 0.45 (moderate)

Using 2009 FIGO classification, MR imaging detected an endometrial cancer of stage IA, IB, II, or IIIA disease in 52 cases (63.5%), 27 cases (32.9%), 2 cases (2.4%), and 1 case (1.2%), respectively.

When considering myometrial invasion using 2009 FIGO classification, among the 52 women with presumed stage IA disease on MR imaging, final histology showed stage IA, IB, II, IIIA disease in 73%, 19.2%, 7.8% and none, respectively. Among the 27 women with presumed stage IB on MR imaging, final histology showed FIGO stage IA, IB, II and IIIA disease in 18.5%, 74%, 3.7% and 3.7%, respectively. Among the two women with pre-operative stage II, final histology showed FIGO stage IA and II in 50% respectively. The only woman with a preoperative stage IIIA had a FIGO stage IB at final histology. Therefore, 59 of the 82 women (70%) were correctly staged by MRI. The correlation between MR imaging staging and final histology was moderate (Kappa= 0.45).

None of the 27 patients with no or less than half myometrial infiltration and grade 1 tumor exhibited positive SN. Three of the 18 patients (16.6%) with no or less than half myometrial infiltration and grade 2-3 tumor had positive SN. Four of the 12 patients (33.3%) with more than half myometrial infiltration and grade 1 tumor had positive SN. Six of 12 patients (50%) with more than half myometrial infiltration and grade 2-3 tumor had positive SN. One of the seven patient (14.3%) with invasion of cervical stroma or serosa of the corpus uteri had a positive SN.

## Discussion

The present study suggests that sentinel node biopsy is an adequate technique to evaluate lymph node status. The use of the 2009 FIGO classification increases the accuracy of MR imaging to stage patients with early stages of endometrial cancer and contributes to clarify the indication of SN biopsy according to tumour grade and histological type.

To our knowledge, the present study is the largest series on SN in endometrial cancer reporting a high SN detection reaching 88.2%. Among the fourteen patients with lymph node metastases one-half were detected by serial sectioning and immunohistochemical analysis. These results are in accordance with those of a recent review of literature on SN in endometrial cancer showing that SN detection rate was depending on sites of injection, technique of labeling, and histological technique for SN analysis [8]. A debate exists on the best site for injections of patent blue and radiocolloid [9,13-18]. Like in the present study, cervical injection has the advantage to be easy and reproducible but exposes to the risk to ignore direct para-aortic drainage. Conversely, injection under hysteroscopic guidance is a more invasive technique and raises the issue whether SN biopsy must reflect tumor or organ lymphatic drainage. Another option could be the use of both dual cervical and colorimetric subserosal injection at the first step of surgery [19]. So far, no trial comparing various injection sites is available to standardize the SN biopsy in patients with endometrial cancer. As in the present study, a higher SN detection rate was noted for technique using dual labeling ranging from 70% to 100% compared to colorimetric or radiocolloid technique alone [8]. Finally, the contribution of serial sectioning and IHC has been proved to detect micrometastases although issues exist on their prognostic relevance [8-10,20-22]. Moreover, previous studies on SN in cervical cancer have underlined the risk of confounding micrometastases with staining of benign inclusions or mesothelial cells [23]. Despite these limitations on the SN biopsy in endometrial cancer, the absence of false-negative rate observed in our experience and in previous studies suggests its relevance in routine practice. This is also supported by the histological validation of the SN biopsy published by Delpech et al [16].

The results of the present study are partly in disagreement with those of two randomized trials showing no benefit for pelvic lymphadenectomy on overall or recurrence-free survival in patients with early endometrial cancer. The ASTEC study showed a hazard ratio (HR) of 1.16 (95% CI 0.87-1.54; p = 0.31) in favor of hysterectomy and bilateral salpingo-oophorectomy without lymphadenectomy and an absolute difference in 5-year overall survival of 1% (95% CI -4 to 6) [6]. With adjustment for baseline characteristics and pathology details, the HR for overall survival was 1.04 (0.74-1.45; p = 0.83) and for recurrence-free survival was 1.25 (0.93-1.66; p = 0.14). However, median follow-up was of only 37 months not allowing proving the positive impact to remove micrometastases. Indeed, a previous study showed that micrometastasis removal in women with endometrial cancer was associated with a significant increase in disease-free survival and that the rate of recurrence reached 35.7% in patients with micrometastases over 40 months of follow-up [10]. In a second randomized trial, Benedetti et al found that both early and late postoperative complications occurred statistically significantly more frequently in patients undergoing pelvic systematic lymphadenectomy but was associated with improved surgical staging with detection of lymph node metastases in 13.3% that is concordant with our rate of metastases in patients with grade 2-3 endometrioid carcinoma and stage IA on MR imaging [24]. However, in these two trials, SN biopsy, serial sectioning and IHC were not performed exposing to underestimate the rate of lymph node metastases and to under-treatment [16].

It is clear that the use of the 2009 FIGO classification with fewer stages may impact on surgical strategy particularly concerning the need for lymphadenectomy. In our experience, none of the patients with grade 1 endometrioid carcinoma and invasion of less than half of the myometrium exhibited positive SN suggesting that lymphadenectomy as well as SN procedure could be omitted. Eltabbakh et al underlined the risk of underestimation for grade 1 endometrioid carcinoma on biopsy with an upstaging on definitive histology in one-third of cases [25]. Hence the combination of histological type and grading on biopsy and presumed MR imaging staging reduce the risk of lymph node misdiagnosis. Our results are in accordance with a recent report using the same protocol for SN biopsy in 42 patients with grade 1 endometrioid carcinoma showing that only 11% of patients with stage I had positive SN [26]. Moreover, as in our experience, the risk of positive non-SN in case of positive SN was 0% for patients with stage I endometrioid carcinoma. This is particularly true when a systematic IHC on SNs is used. Indeed, Altgassen et al demonstrated that when SNs were IHC stained, the sensitivity of SN biopsy rose to 83.3% with a NPV of 93.8% [27]. Although recent trials underlined the low relevance of systematic lymphadenectomy in patients with grade 2-3 endometrioid carcinoma and invasion of less than half of the myometrium, our relative high incidence of positive SN justify its use. Indeed, for these patients, our rate of positive SN reached 16.6% with an incidence of micrometastases of 50% addressing the issue on the evaluation of lymph node status. Moreover, all positive SNs were found in patients with grade 2 endometrioid carcinoma suggesting that a cut-off exists between grade 1 and grade 2-3 endometrioid carcinoma. These results are supported by a recent review of Bernardini & Murphy addressing issue on both the use of the SN technique and the consideration of a binary grading system to simplify triaging of patients [28]. For patients with invasion equal to or more than half of the myometrium whatever the tumor grade, the rate of positive SN was 31.6%. Moreover, the rate of positive SN was 14.3% for grade 1 and invasion equal to or more than half of the myometrium underlining that evaluation of lymph node status cannot be omitted. For patients with non-endometrioid carcinoma, the sample size was too low to draw conclusions but for stage IA non endometrioid carcinoma whatever tumor grade, the rate of positive SN was 10% and reached 44.4% for stage IB. These results are in accordance with those of Mariani et al recommending systematic pelvic and para-aortic lymphadenectomy in non-endometrioid carcinoma [29].

The main issue for women with endometrial cancer is to assess the presumed FIGO stage to determine surgical management. In addition to preoperative endometrial biopsy, MR imaging is the main admitted imaging technique to stage endometrial cancer. In our experience, using the 1988 FIGO classification, only one-third of patients were adequately staged. Misdiagnosis occurred mainly for patients with 1988 stage IA carcinoma hence the use of the 2009 FIGO classification merging stage IA and IB contributes to increase the accuracy of MR imaging. Despite these modifications, the accuracy of MR imaging to determine myometrial invasion remained relatively moderate. Hence, these results reinforced the potential relevance of SN biopsy. Previous meta-analysis on MRI have demonstrated a high overall performance of MRI reaching 0.87 (95% CI: 0.85-0.89) with a performance for myometrial and cervical invasion of 0.83 (95% CI: 0.79-0.87) and 0.92 (95% CI: 0.87-0.95), respectively [30]. The discrepancies between our results and those of the meta-analysis are probably linked to the inclusion in our study of patients with early stages of disease while the meta-analysis included mainly patients with 1988 stage IB, IC and IIIa.

Some limits of the present study have to be underlined. First, although the present study is the largest series, our incidence of patients with lymph node metastasis was too low to build a nomogramme to identify patients that could benefit of the SN biopsy. However, the low accuracy of imaging techniques such as MR imaging and PET-FDG reinforces the potential relevance of SN especially in case of endometrial cancer affecting elderly women often obese and with co-morbidities. Second, in accordance with French guidelines, except for clear cells and serous papillary carcinomas, no systematic para-aortic lymphadenectomy was performed. This could represent a potential cause of false negative rate underestimation. Third, the absence of consensus on SN technique rends comparison difficult between series. We opted for a dual cervical injection thank to its easiness and reproducibility although some para-aortic involvement could be missed by ignoring direct para-aortic drainage impacting on both SN detection and false negative rate. However, previous studies have emphasized on the low incidence of isolated para-aortic metastases [31]. Abu-Rustum et al in a large series of endometrial cancer reported that only 1.6% had positive paraaortic nodes with negative pelvic nodes. Moreover, among the 187 patients with a final diagnosis of grade 1 endometrial cancer, only 2 (1%) had a positive paraaortic node with negative pelvic nodes [32]. Fourth, in the present study, the comparison of the two FIGO classifications mainly based on myometrial involvement could be a potential bias by ignoring the impact of lymph node status. Moreover, the role of MRI to assess FIGO classification of women with endometrial cancer remains a matter of debate. However, in a review of the literature, Selman et al underline that among the various imaging techniques available to determine endometrial cancer stage, MRI appears the best option. Finally, we did not used a systematic CAM immunostaining to differentiate true micrometastases from mesothelial staining that could overestimate the rate of lymph node metastases [33].

In conclusion, our results suggest the relevance of SN biopsy to determine lymph node status in patients with endometrial cancer. The combination of tumor grade, histological type and features of MR imaging associated to the use of the 2009 FIGO classification of endometrial cancer contribute to clarify the good candidates for SN biopsy.

## Competing interests

The authors declare that they have no competing interests.

## Authors' contributions

MB has made substantial contributions to conception and design, or acquisition of data, or analysis and interpretation of data; he has been involved in drafting the manuscript or revising it critically for important intellectual content. MK collected data and has been involved in drafting the manuscript. CC collected data and has been involved in drafting the manuscript. EC collected data and has been involved in drafting the manuscript. JS collected data and has been involved in drafting the manuscript. RR has made substantial contributions to conception and design, or acquisition of data, or analysis and interpretation of data. ED has been involved in drafting the manuscript or revising it critically for important intellectual content. All authors have given final approval of the version to be published.

## Pre-publication history

The pre-publication history for this paper can be accessed here:

http://www.biomedcentral.com/1471-2407/10/465/prepub
